# The Diagnostic Importance of the Leukocytes in the Blood

**Published:** 1902-05

**Authors:** J. M. Thomas

**Affiliations:** Griffin, Ga.


### THE DIAGNOSTIC IMPORTANCE OF THE LEUKOCYTES IN THE BLOOD.*

By J. M. THOMAS, M.D.,

GRIFFIN, GA.

That the condition of the blood has something to do with the outbreak, developing and prognosis of diseases, is a fact already noted by the great physicians of ancient times.

Hippocrates, the father of medicine, speaks about a bad mixture of blood which causes, in certain individuals, diseases which are not amenable to treatment.

Celsus, the great Roman surgeon, has written twenty volumes about the origin and treatment of diseases, and has devoted a whole chapter to "thick blood" and "thin blood" causing all kinds of diseases.

Even the great physicians who lived in the beginning of the last century, among them Malgaigne, the great surgeon of Napoleon I, mentions the great influence of the blood on the prognosis of healing of wounds. Malgaigne, who was one of the greatest war surgeons who ever lived, was present in all the Napoleonic battles, states, as he describes the wounded soldiers after the battle of Jena, that one-third of the wounded French soldiers were not to be saved by any art of treatment, for the condition of their blood was such that they could not stand operations and treatment.

After the invention of the microscope by Jansen in 1590, but

*Read before the Georgia Medical Association, April 18, 1902.

little attention was paid to the examination of the blood, or to the examination of the human tissues. The first scientific histological study was done by Schwann in 1839; who studied at first the plants, and pointed out that they consist of cells and have a similar structure to the human tissue. He discovered the cell and made, at first, the statement that all living organisms, plants or animals, with all their complicated organs, originate from small bodies, which can be detected under the microscope, and which he called "cells," containing a ground substance, the "protoplasma" in which the whole living process takes place. At this time the second, and also important part of every cell, which always must be present, the "nucleus," was not yet discovered.

His follower was Virchow, the greatest German scientist at present, who wrote his celebrated book about the "cellularpathology," in which he claimed that in order to find out the cause of pathological conditions, the cells, which compose the tissues and the organs, have to be examined and their changes have to be studied and microscopically recognized. This theory first met with great opposition because it was in direct contrast with the general views of the scientists, who mostly were inclined to favor "Humoral-pathology," which claimed that the fluids in the system, the lymph, and in the second place, the mixture of the blood play the greater role in the pathogenesis of the diseases. But the great discoveries which have been made by the microscope, especially the study of benign and malignant tumors, founded by Virchow, soon gave a black eye to the followers of the Humoral Pathology.

The investigation about the inflammation made by Virchow's pupil, Kohnheim, and the study of the microscopical appearance of the blood by Brücke have been great supports for Virchow's views.

Brücke, of Vienna, (the greatest physiologist of the last century, and the pupil of Johannes Müller) made his classical investigations about red and white blood corpuscles, about the coagulation of the blood in vessels, the walls of which are not normal; and his pupil, Rollett, in Gratz, Austria, devoted his time to investigate the process of digestion in the stomach, and made the sensational discovery about the action of the hydrochloric acid and the pepsin.

Virchow discovered, in 1845, leukemia. He examined the blood of a patient who was suffering from a general swelling of the lymphatic glands, and he found an immense increase of the white blood corpuscles, and said: "I see here white blood," and called the condition white blood disease (translated, in Greek Leukemia), and as he demonstrated the case in the medical society he said: "I think the leukocytes will play soon a great role in the pathology, as well as in the diagnosis, of diseases."

Shortly afterwards, as already mentioned, his pupil, Kohnheim, founded his "inflammation theory," which is based on the fact that every inflammation is characterized by a diapedesis of the red blood corpuscles through the capillary walls by an immigration of the leukocytes. These immigrated leukocytes form the product of inflammation, the exudate. To-day, we know that such an exudate may be serous, fibrinous, sero-fibrinous or purulent, or tuberculous, depending on the infection either with staphylococci, pneumococci or streptococci or tubercular bacilli.

The improvement of the lenses in the microscope has also favored very much the study of the blood, and Max Schultze, in 1870, was able to find out that the white blood corpuscles in the blood are of different kinds. He described the cells containing one nucleus and those which contain two or more nuclei, and he also pointed out at first, that some leukocytes contain granules in their protoplasm, while others do not.

Thoma suggested the apparatus for counting the red and white blood corpuscles and the clinicians found out that in anemic conditions the red corpuscles were diminished, while the leukocytes were sometimes increased in number.

Gowers paid first attention to the condition of the hemoglobin, and was able to show, with a hemoglobinometer, which he constructed, that the percentage of hemoglobin in the normal blood was 100 per cent., while in anemic conditions it might be diminished to 15 per cent.

Many apparatus were invented, but the best, which is now used everywhere for scientific purposes, is that by Von Fleischel, of Vienna.

The great discoveries in the bacteriological line made by Pasteur, who discovered the anthrax bacillus, by Koch, who, in 1881, discovered the tubercle bacillus, have somewhat turned off the interest of the profession from the examination of the blood, especially since the technique was very complicated. A sensational discovery in the blood, which was made on the 8th of November, 1880, was not known to the public immediately, and from this moment we can date the beginning of the scientific hematology.

Laveran, a French military surgeon, discovered in the bood the specific germ of malaria, the plasmodium. He lived with his troops in Algiers, in a French Province, a country where malaria is very frequent, and he examined the blood of the soldiers who were suffering from the fever. He took a drop of blood, spread it out on a slide, covered it with a red cover-glass and found within the red corpuscles hyaline bodies, with an ameboid movement, and within these bodies he found black pigment granules, also moving. He said immediately that these were living organisms, and after he had convinced himself that these organisms were present in all cases examined by him, he wrote a lengthy paper on the subject, dedicated to the Academy of Science, in Paris. But even in his native country but very little attention was paid to this subject, and in Germany and Italy no one took any notice of his publication, and it was only in years afterward that he obtained any degree of fame.

It is often the case that a young and struggling scientist who discovers something and presents it to the world, has his young ardor and enthusiasm considerably dampened, when he should be encouraged, only to have the scientific world wake up in after years to the fact that his discovery was a genuine benefit.

After Laveran acquired more experience on this subject he went to Italy to find out whether in that country, where malarial conditions generally exist, if the same germ could be found in the blood there. After he convinced himself that this was the case, he took opportunity to read a paper on this subject before the medical society in Rome, but met with great opposition there, because there was another opinion prevailing in regard to malaria at that time.

As has been before mentioned, this was about the time of the great discovery of the bacilli, as the origin of infectious diseases, and at this time the general opinion was that every disease with fever must be due to a bacillus. Tomasi Crudelli, pathologist at the University of Rome, had discovered a bacillus in the blood of malarial patients, which he called the "Bacillus of Malaria;" but later investigations have proven that he was mistaken, for this bacillus was an artificial production.

He collected the blood in small glass capillaries and closed both ends by heating them by flame, in order to preserve the blood for microscopical investigation in his laboratory. For this he needed a high temperature to bring the ends of the capillaries to the point of melting, and by exposing the blood to such a high temperature he produced a coagulation and destruction of many blood corpuscles. When he now examined the blood under the microscope he found rod shaped elements, resembling very much bacilli, but which were only parts of destroyed red blood corpuscles.

In 1884 Marchaifava, pathologist in Rome, and his assistant Celli, examined the blood of malarial patients by staining it with methylenblue. They found within the red blood corpuscles blue bodies, containing black pigment granules. Marchaifava interpreted very correctly these black bodies being melanin (destroyed hemoglobin), but he concluded wrongly that these black bodies were the cause or the disease. He claimed that in malaria a degeneration of the red blood corpuscles takes place, that their hemoglobin is changed into black granules, and that this degeneration of the hemoglobin causes the fever. He took the sequence for the cause. He called his theory "degeneration theory," and at the International Medical Congress at Copenhagen, in 1885, he demonstrated his specimens and made a great speech about his discovery, which met with great interest and acknowledgement, for Marchaifava was known as a great authority on microscopy.

But a year later he found out his mistake, when he took a drop of fresh blood and examined it under the microscope. He found, without any difficulty, hyaline ameboid bodies present within the red blood corpuscles, and within these bodies he found his black pigment granules. In other words, he found the same thing Laveran had seen and described five years ago. Nevertheless, he claims for himself the priority of this discovery, and from this time on a great paper war began between Italy and France on this subject. Every unprejudiced observer who had the opportunity to study the subject will give the credit of this discovery to Laveran, who has not only discovered the plasmodium with his microscope of much simpler construction than ours now, but also he described the germs in such an extensive manner, that to-day we have only a little to add to his observations. But the great merit of having completed the subject, especially from the clinical standpoint, of having discovered the special forms of the malarial plasmodia and their connection with the special types of the fever, as well as the connection between the development of the germs in the blood and in the inner organs and the outbreak of the chill, this merit undoubtedly belongs to the Italian school. In the first place to Golgi, then to Marchaifava, Celli, Bignani, and last, but not least, to some men in our own country, to Osler, his pupil Thayer, and to Dock, now professor at Ann Arbor.

After this great discovery became universally known, not only the pathologists and microscopists, but also the clinicians got hold of it, and everywhere, where medicine was taught, great attention was paid to the examination of the blood. But as long as the methods of preparing the specimens were very complicated, only the scientific world could devote their time to the subject.

A regenerator in hematology was Ehrlich, from Berlin, now everywhere known as one of the men connected with the discovery of antitoxin in diphtheria. He devoted five years of his life (from 1887 to 1892) to the study of the blood, and has improved the technique greatly; so much so that we could call him the father of the modern clinical hematology.

Ehrlich, together with Koch, improved the methods of the staining of tubercle bacilli, and were first to suggest the fixation of blood specimens by slow heat, for the former ways of drawing the specimens through a flame, as we generally do it with bacterial specimens, hinders the staining process by the coagulation of the albuminous substances in the blood, which, by this method, their staining properties are almost entirely lost.

Ehrlich fixed his blood specimens by heating them on a copper plate, which was divided in four parts, and which was heated by a flame beneath one end of the plate. First he placed his specimens for a half of an hour in the furthest area, and every half hour he placed them in an area nearer to the flame, so that the whole fixing process took place gradually and the specimens were not immediately exposed to a very high temperature. This method, which took him full two hours, is now accomplished within five minutes, as I will explain later on.

Ehrlich suggested further the "Eosin" stain as the best stain for the hemoglobin and the hemotoxyline, and the methylenblue as stains for the nuclei. He introduced the so-called "Triple stain," also called "Ehrlich stain." This is the best stain for quick diagnostic purposes, for, as we will see later on, the whole staining process does not take longer than one-half to one minute.

Not only the technique of the hematology (thanks to Ehrlich for its improvement and, to a certain extent, its foundation), but he also made very valuable and original investigations about leukemia and anemic conditions. He discovered the quality of certain granules within leukocytes to take stains of a certain chemical condition, acid and basic, or neutral. He first described the myolocytes, cells which I will show you later only occur in the blood in certain diseases and are of greatest diagnostic importance.

Ehrlich also studied and described the so-called nucleated red blood corpuscle, and stated that they occur in two varieties in the blood, as normoblasts and as megaloblasts. But he first paid more attention to the histological part of hematology than to the clinical significance.

It is the great merit of one of the younger members of the Vienna school, of the present chief of the internal clinic at the University of Vienna, of Hofrath Neusser, to have founded the clinical side of hematology in relation to diagnosis, differential diagnosis and prognosis at the bedside, and to have simplified the methods of examination, so that we are now able within ten minutes to make a complete examination of the blood for diagnostic purposes.

Neusser, the greatest diagnostician in the Vienna school, began to study the blood in 1888, and is now considered the greatest authority on this subject in Vienna. I had opportunity to study the blood with him and with one of his assistants during my stay in Vienna, and will try in this paper to give the principal points I got there, and which I have used in my practice since my return from Vienna.

Before I enter into the very subject of this paper I must give credit to the authorities in our own country, who have also done great work in this line, as the already mentioned Osler, Dock, Thayer, and last, but not least, to Cabot, of Boston, who has written a very good book on the blood.

The subject of hematology has grown so rapidly within the last ten years, and the literature upon this subject is so great, that I can only take a chapter of it and speak about it today.

Virchow was quite right when he said, in 1845, that "the lueko-cytes will play a great role in the diagnosis and prognosis of diseases." First, I will say a few words about the technique of making blood specimens. It is absolutely necessary to make blood specimens in a proper manner, otherwise it is quite impossible to draw any conclusions from them. The cover-glass must be cleaned thoroughly with alcohol and ether, and the skin from the lobe of the ear must be cleaned in the same manner. The lobe of the ear is the best place from which the blood should be obtained, because the least pressure is necessary to obtain a few drops of blood on account of the richness of the blood in this part of the body. In anemic conditions it is often very difficult to get a few drops of blood from the finger without using great pressure. By this pressure more fluid is pressed out, therefore the drop of blood is more diluted; then, by pressing, more leukocytes are pressed out, because they are more movable than the red blood corpuscles, and finally pressure injures the red blood corpuscles, changing their shape and often producing a crinated appearance of them. From all this follows that the lobe of the ear is more preferable, because the prick is less painful than in the point of the finger; and, finally, the patient does not see what happens, which is very necessary, especially in hysterical women and children.

Then spread out the drop of blood between two cover glasses by drawing them apart and using a little forceps in order to separate them; then let them dry in the air for a few minutes, and then fix them in a little copper stove by heating them to 240 degrees F. This takes about four or five minutes. For quick diagnostic work it is sufficient to fix the specimens by holding for a few minutes above a flame. After that the specimen is to be stained. Every staining is a chemical process.

The protoplasm in all tissues, in the blood it is the hemoglobin, has a basic reaction, it therefore takes the acid stain; while the nucleus has an acid reaction and consequently takes the basic stain. For staining of blood specimens two methods are used: the double stain and the triple stain. The latter is the quicker method, and the best way of staining for diagnostic purposes in office practice.

The stains consist of orange G., which stains the hemoglobin yellowish brown; of methylgreen, which stains the nuclei blue; and of acidfuchsin, which stains the granules of some leukocytes.

The technique of using the stains is very simple. You place a few drops of this stain on the specimen, and half a minute later you wash it out in ordinary water, dry between filter paper, and mount in Canada balsam. Within ten minutes it is possible to have a properly stained specimen under the microscope, one minute spreading out, two to three minutes drying in the air, four to five minutes fixation, one to two minutes staining and mounting.

The double stain on the other hand, which consists of eosin and hematoxylin, or of the combination of eosin and methylenblue, gives nicer pictures, and is more for demonstration of blood specimens; the staining takes from fifteen to twenty minutes.

For examination of blood specimens we need to have a good microscope, with a so-called one-twelfth oil immersion.

The blood consists of red and white blood corpuscles. The red corpuscles are normally in a greater amount present than the white; in one cubic millimeter of blood we find five millions of red blood corpuscles, and only eight to ten thousand leukocytes. The leukocytes may show one nucleus, we call them mononuclear, or they may contain two or more nuclei; then they are called polynuclear. Some of them contain granules in their protoplasm, while others are homogeneous, so that with our best lenses, we are not able to find any structure.

About 64 per cent. of the leukocytes in the normal blood are polynucleated, and show, if stained with the triple stain, small violet granules, which are called "neutrophile," because they take acid and basic stains. Large granulated leukocytes are very scarce in the blood. These granules take only acid stains, and are called "acidophile," and since the first acid stains with which Ehrlich (who described them first) has stained them, was eosin, it is now customary among the hæmotologists to call these cells eosinophiles.

The mononucleated leukocytes are normally in a smaller amount present than the polynucleated; the relation is about two to one. There are three forms of mononucleated leukocytes present: the small mononucleated, which take their origin in the lymphatic glands are called lymphocytes, they have a large nucleus, but very little protoplasm; the large mononucleated, taking their origin in the spleen, have a large light colored nucleus and more protoplasm than the lymphocytes.

The transitional cells have a nucleus, which is either kidney, horse-shoe or embryo shaped. They are an intermediate stage between the mononucleated and polynucleated. These are the oldest cells and take their origin from the mononucleated, by a process of transformation taking place within the nucleus.

The age of the nucleus can be recognized by its color, the older the nucleus, the darker its color.

When we examine fresh blood without staining we see that the leukocytes show a slow ameboid movement. For this purpose it is necessary to use a so-called "heated stage" where the temperature of the body must be attained, and where we must examine a cell for hours. There we find the leukocytes slowly changing their place and surrounding foreign bodies and carry them away in the inner organs. This peculiarity of the leukocytes is called phagocytosis, and was first discovered by Metchnikoff, the great pupil of Pasteur.

Metchnikoff was able to prove by very ingeniously arranged experiments, that the leukocytes not only carry away foreign bodies and destroyed elements as malarial pigment, but that they also take up toxic agents present in the circulation in infectious diseases.

We will have opportunity to go into details about this interesting subject.

There are conditions in which the number of the leukocytes are increased, this is called a "leukocytosis"; and they also may be diminished in number, this is called "leukopenia."

An increase of the leukocytes is a valuable diagnostic and prognostic symptom in acute infectious diseases, and it is also of great significance in anemic conditions. The decrease of the leukocytes, the leukopenia, is less frequent; it is only found in a few conditions, chiefly in typhoid fever and in primary pernicious anemia.

About the origin of leukocytes there are many theories; the most of them are founded by experiments on animals. Certain chemical agents, toxines of bacteria, ptomains, and certain drugs, have the quality of increasing the leukocytes in the peripheral circulation by attracting them. This attractive power is called chemotosis; it can be positive or negative.

The diplococcus, the specific germ of croupous pneumonia, has an attractive power over the leukocytes, while the typhoid bacillus expels them in the inner organs, this is called negative chemotosis. In pneumonia, therefore, the leukocytes are increased in number, while in typhoid fever there is a leukopenia.

Certain drugs, as quinine, strichnine, and all the antipyretics, as well as cold baths, increase the leukocytes. These facts are very important in regard to acute infectious diseases. Our modern theory is, that the fever is a reaction of the organism against the infection, and that the serum of the blood acts against the toxic agents produced by the bacteria. The leukocytes, which as "phogocytes" carry away the toxic agents from the blood, act as soldiers fighting against the infection. The more leukocytes present the greater the opportunity of victory over the enemy. Cases of pneumonia with a great leukocytosis generally give a better prognosis than cases without, or with a slight one. The use of antipyretics in such cases is therefore not only indicated for the purpose of reducing the temperature for a few hours, but also in order to increase the leukocytes.

Quinine, which acts as a specific in malaria, by destroying the plasmodia themselves, also increases the number of the leukocytes, which are diminished during malarial fever. In typhoid fever the leukocytes are diminished, this diminution, already occurring at the end of the first week, just the time when the majority of cases come under the control of the physician, is a valuable differential diagnostic point between typhoid and other acute and infectious diseases.

Cold baths have a splendid effect on the course of the disease, surely not by reducing the temperature for a few hours, but by increasing the respiration and also by producing a temporary leukocytosis. It is the great merit of Thayer, from Johns Hopkins, to have discovered this, by observing a great number of cases of typhoid in this respect.

The presence or absence of a leukocytosis is also a valuable point in patients who have been operated upon to find out whether there is any sepsis threatening or not, for the absence of a leukocytosis points against sepsis, while its presence makes it very probable.

If there are suppurations in the system, as in the brain, the nose, the pleura, the lungs, the peritoneal cavity, the liver, the appendix, and in the joints, a leukocytosis with the appearance of peptone in the urine can always be noted, if the exudate is purulent; while, if it is tubercular, neither peptone nor a leukocytosis can be found.

But a leukocytosis may occur in different types. This leukocytosis I have spoken of is the acute inflammatory. It is an increase of the polynuclear leukocytes associated with normal conditions of the red blood corpuscles. In anemic conditions following carcinoma, syphilis, and severe hemorrhages, there is also a polynuclear leukocytosis present, but it is associated with alterations of the red blood corpuscles. Such a condition of the blood is called secondary anemia.

Of greatest clinical significance is an increase of the small mononucleated leukocytes, of the so-called lymphocytes, which take their origin from the lymphatic glands. There is only one disease in which this condition of the blood is found, and this is "lymphatic leukemia" characterized by a general swelling of the lymphatic glands. This disease always ends fatally within a few months, and it is of greatest importance for the physicians to make an early diagnosis in order to protect himself, for the disease in the beginning makes no trouble and might easily be taken for scrophulosis. Therefore if a physician, who does not know anything about the examination of the blood, sees such a case, and takes it for scrophulosis and makes a good prognosis and in one or two months afterwards the patient dies, he does not increase with this his reputation.

A doctor who is familiar with the examination of the blood makes the diagnosis within a few minutes and is able to tell immediately that there is a very dangerous condition, fatal within a short time.

The changes in the blood in lymphatic leukemia are so striking that no mistake is possible.

Another condition of the blood, called splenic leukemia, is of greatest importance for the surgeon and gynecologist. It is characterized by an immense enlargement of the spleen, which almost fills the whole abdomen and pelvis, and may be confounded with a tumor in the abdomen and could be taken for a growth to be operated on. Even an exploratory laparotomy, to get an idea about the connection of the tumors with the other organs, as it is often done, could be extremely dangerous, for the patient could die on the table on account of thrombosis of the heart, which is very frequent in leukemia.

In the blood of splenic leukemia might be an immense increase of the leukocytes, while normally the relation between red and white is 500 to 1; in this form of leukemia it might be 10 to 1, 5 to 1, 2 to 1, 1 to 1, 1 to 2; this means twice as many white as red blood corpuscles. This great increase of the leukocytes favors the coagulation of the blood and gives rise to thrombosis of blood vessels, and under an anesthetic a thrombosis of the largest blood vessel might occur. The number of such cases recorded who have died on the table is not by any means small.

To-day every surgeon, if he has to do with an abdominal tumor, the nature of which is not quite clear, examines first the blood in order to exclude a splenic leukemia before he operates.

Together with an enlargement of the spleen, there is also very often an affection of the bone marrow present, especially in the sternum, ribs, vertebra, and also in the long bones. The patients complain of pains in the bones. If these pains are present in the night the case could be taken for syphilis, because nocturnal pains in the bones are very frequent in syphilis. Such a mistake could be quite dangerous for the patient, because if, in such a case, an anti-syphilitic treatment, an inunction cure, should be applied, the leukemic condition with the anemia would get worse, because mercury itself produces anemia.

The condition of the blood in this form of leukemia is very characteristic: there is a mixed leukocytosis present. All varieties of leukocytes are found—mononucleated, polynucleated, granulated and non-granulated. In order to make the diagnosis of a leukemia it is necessary to examine the blood, because without it, and from the clinical symptoms alone, it is impossible to make the differential diagnosis.

The so-called large granulated or esinophile cells, which appear in the normal blood in a very small proportion, only from one to three per cent., also play an important diagnostic and prognostic role. In all acute infectious diseases, during the time of the fever, they disappear from the blood and reappear when the fever subsides, only in malaria and scarlet fever they are present during the time of the fever. In pneumonia they reappear before the fever subsides, either on the day of the crisis or on the day before the crisis. This reappearance is a favorable symptom, pointing with certainty to the coming crisis, even if the patient still has high fever.

Every physician who examines the blood of his pneumonia patients on the seventh or eighth day of the disease knows what it means when, twenty-four hours before the fall of the temperature, by the finding of the esinophile cells in the blood.

I am able to predict with absolute certainty the fall of the temperature, and with it the favorable change in the course of the disease. After the crisis has taken place we always find the esinophiles present; their absence in the blood after the fall of the temperature is a bad symptom, either pointing to a pseudocrisis or even to a fatal termination, for the absence of these cells shows that the resolution of the exudate in the lungs has not yet taken place.

I have tried to show in this paper that the condition of the leukocytes in the blood is of greatest importance in the diagnosis and prognosis of diseases. I hope the few points in this paper, which I have tried to make clear, will convince you more thoroughly that hematology is a very important branch of general medicine, and that every intelligent physician should, at least in a general way, be able to make a scientific examination of the blood.

I hope I will later on have opportunity to tell you more about this subject, especially concerning the significance of the red blood corpuscles.

